# Assessing Executive Abilities Following Acute Stroke with the Trail Making Test and Digit Span

**DOI:** 10.3233/BEN-2011-0328

**Published:** 2011-08-29

**Authors:** Elaine Tamez, Joel Myerson, Lucy Morris, Desirée A. White, Carolyn Baum, Lisa Tabor Connor

**Affiliations:** ^1^Department of PsychologyWashington UniversitySaint LouisMOUSA; ^2^Program in Occupational TherapyWashington University School of MedicineSaint LouisMOUSA; ^3^Department of NeurologyWashington University School of MedicineSaint LouisMOUSA; ^4^Department of RadiologyWashington University School of MedicineSaint LouisMOUSA

**Keywords:** Digit span, trail making test, stroke, executive function

## Abstract

The Trail Making Test and Digit Span are neuropsychological tests widely used to assess executive abilities following stroke. The Trails B and Digits Backward conditions of these tests are thought to be more sensitive to executive impairment related to frontal lobe dysfunction than the Trails A and Digits Forward conditions. Trails B and Digits Backward are also thought to be more sensitive to brain damage in general. Data from the Stroke and Lesion Registry maintained by the Washington University Cognitive Rehabilitation Research Group were analyzed to compare the effects of frontal versus nonfrontal strokes and to assess the effects of stroke severity. Results showed that the performance of patients with frontal and nonfrontal strokes was comparable in each condition of both the Trail Making Test and Digit Span, providing no support for the widely held belief that Trails B and Digits Backward are more sensitive to frontal lobe damage. Further, Trails A was as strongly correlated with stroke severity as Trails B, whereas Digits Backward was more strongly correlated with stroke severity than Digits Forward. Overall, the Trail Making Test and Digit Span are sensitive to brain damage but do not differentiate between patients with frontal versus nonfrontal stroke.

## 1. Introduction

The Trail Making Test and Digit Span are neuropsychological tests widely used to assess executive abilities in patients with stroke [1]. Each test includes two conditions, with the Trail Making Test comprising Trails A and Trails B and Digit Span comprising Digits Forward and Digits Backward. Performance on Trails A and Digits Forward is thought to reflect basic abilities such as motor speed in the case of Trails A and attention span or temporary storage capacity in the case of Digits Forward. Performance on Trails B and Digits Backward, however, is thought to reflect higher-order executive abilities [2,3]. Because executive abilities are largely subserved by the frontal lobes [4,5], it is not surprising that the purported executive conditions of these tests (i.e., Trails B and Digits Backward) are considered to be more sensitive to frontal lobe damage [4, 6].

During the Trails A condition of the Trail Making Test, participants are given a sheet of paper with circled numbers (from 1–25) and are asked to connect the circles in numerical order as quickly as possible [7]. During Trails B, participants are given a sheet of paper with circled numbers and letters and are asked to connect the numbers and letters in order, alternating between numbers and letters. Trails B is viewed as relying more on executive control because it not only requires the abilities used for Trails A (e.g., visual scanning and motor speed) but also requires alternation between letters and numbers, presumably involving the executive abilities of set shifting and cognitive flexibility [7–10].

ISSN 0953-4180/11/$27.50 © 2011 – IOS Press and the authors. All rights reserved

Functional neuroimaging in healthy adults has shown that performance on Trails B is associated with greater frontal lobe activation than performance on Trails A [11], and it is widely believed that focal damage to the frontal lobes affects performance on Trails B more than Trails A (e.g. [12]). Research, however, does not support this claim. In fact, any differences between patients with frontal and nonfrontal damage are found on Trails A, the purported non-executive condition of the test ([13], for a meta-analysis see [14]), with slower performance by patients with nonfrontal than frontal damage. Of particular importance to the current study, similar findings have been reported in patients with stroke affecting frontal and nonfrontal brain regions [15]. Taken together, these results question the utility of Trails B as a specific marker of frontal lobe damage.

Like the Trail Making Test, the Digit Span subtest of the Wechsler Memory Scale comprises two conditions, Digits Forward and Backward. Digits Forward is the simpler and less demanding condition requiring basic cognitive skills, whereas Digits Backward is the more complex and demanding condition requiring executive abilities [16,17]. The view that Digits Backward relies more on executive abilities than Digits Forward is supported by the factor analytic results of Glisky et al. [2], who found that Digits Backward loaded onto a "frontal factor" along with other neuropsychological tests such as the Wisconsin Card Sorting Task and verbal fluency [2]. In addition, in neuroimaging studies with healthy adults, performance on Digits Backward has been linked to greater frontal activation than performance on Digits Forward [18]. Accordingly, Digits Backward is considered to be a better measure of executive aspects of working memory [10,19]. It should be noted, however, that the manipulation of information during working memory tasks such as Digits Backward also requires activation of posterior brain regions (e.g., superior and inferior parietal cortex, superior temporal cortex), suggesting a role for nonfrontal brain regions as well [18,20].

In addition to being used as markers of frontal brain damage, Trails B and Digits Backward are thought to be particularly sensitive to brain damage in general [7–9,16]. This assumption, however, has been challenged. For example, Wilde et al. [21] examined Digit Span performance in various clinical groups (for details see technical manuals for Wechsler Memory Scale III and Wechsler Adult Intelligence Scale III). Performance was compared with that of demographically-matched controls from the WMS-III standardization sample [21]. Notably, only four of twelve clinical groups displayed impaired performance on Digits Backward, and three of these groups displayed impaired performance on Digits Forward as well. Only the Alzheimer disease group demonstrated an impairment specific to Digits Backward. Overall, these findings provide little support for the notion that Digits Backward is more sensitive to brain damage in general than Digits Forward. It should be noted, however, that patients with stroke were not examined in the study by Wilde et al. [21].

In the current study, we examined the effects of stroke on Trail Making Test and Digit Span performance. We specifically sought to determine whether performance on Trails B and Digits Backward is more impaired in patients with frontal than nonfrontal brain lesions. In addition, we sought to determine whether performance on Trails B and Digits Backward is more impaired than performance on Trails A and Digits Forward as stroke severity increases, regardless of lesion location.

## 2. Method

### 2.1. Participants

The data reported here were from the Stroke and Lesion Registry maintained by the Cognitive Rehabilitation Research Group (CRRG) in the Occupational Therapy Program at the Washington University School of Medicine. Patients from the Registry who were included in this analysis ($n = 5565$) were admitted to Barnes-Jewish Hospital in Saint Louis between 2003 and 2006 and were seen by the Stroke Management and Rehabilitation Team. Patients received a cognitive assessment that included the Trail Making Test and Digit Span within the first 72 hours of their hospital stay. The National Institutes of Health Stroke Scale (NIHSS) [22] was administered during this time and provided an assessment of stroke severity based on neurological impairment. Over the history of the registry, 78 percent of patients explicitly consented to have their data used for research. Some of the patients seen at the hospital were not consented because they were discharged prior to being seen or were too sick to be approached.

Of the total CRRG sample consented, individuals with a history of chronic depression, dementia, drug or alcohol abuse, kidney dialysis, schizophrenia, or sickle cell disease were excluded from our analyses, leaving 3789 patients. Patients with no record of a cognitive assessment or no data regarding lesion location were then excluded, bringing the sample to 856. Participants younger than 40 years of age or older than 90 years of age were also excluded, which reduced the sample to 689 patients. Because not every patient completed both the Trail Making Test and Digit Span, final sample size and characteristics for each test are reported separately following a description of lesion location.

### 2.1.1. *Lesion location*

Patients were classified as having frontal or nonfrontal lesions based on findings from either computerized tomography (CT) or magnetic resonance imaging (MRI) with MRI sequences including DWI, T2, and FLAIR. Lesions were classified using the vascular templates developed by Damasio and Damasio [23], based on film and clinical neuroradiological reports. In no case were T2 or FLAIR hyperintensities considered infarctions if not mentioned in the radiological report. Frontal patients had lesions in superficial vascular territories supplied by the orbito-frontal, prefrontal, pre-central, and central branches of the middle cerebral artery (MCA) or in the deep subcortical white matter or anterior branch of the anterior cerebral artery (ACA) vascular distribution adjacent to this MCA region. These territories encompassed all of the frontal lobes as well as the postcentral gyri and underlying white matter of the anterior parietal lobes. Nonfrontal patients had lesions in any other brain region. There were a number of patients who could not be classified as either frontal or nonfrontal because they had lesions in both regions or because they had no discernible old or new lesions. In addition, because lesion location information was recorded for no more than three lesions per patient (with acute lesions prioritized over chronic lesions when multiple lesions were present), patients with more than three lesions were not classified and their data were not included in analyses.

### 2.1.2. *Trail making sample*

To examine whether Trails B better differentiated frontal and nonfrontal stroke groups than Trails A, we excluded data for 95 patients because they had both frontal and nonfrontal lesions. In addition, we excluded data for 15 frontal and 37 nonfrontal patients because they had four or more lesions. After these exclusions, data were available for 45 frontal and 122 nonfrontal patients. Frontal and nonfrontal groups were similar in years of age (frontal: $M = 63.5$, $SD = 12.8$; nonfrontal: $M = 65.2$, $SD = 11.6$; $t\ (164) = 0.84$, $p > 0.05$), years of education (frontal: $M = 13.3$, $SD = 9.7$; nonfrontal $M = 12.3$, $SD = 2.4$; $t\ (162) = 1.65$, $p > 0.05$), gender (frontal: 51.1% male: nonfrontal: 54.1% male: $\chi^2(1) = 0.12$, $p > 0.05$), and ethnicity (frontal: 58.2% Caucasian; nonfrontal: 62.2% Caucasian: $\chi^2(4) = 4.35$, $p > 0.05$).

To examine the relationship between stroke severity (i.e., NIHSS score) and Trail Making Test performance regardless of lesion location, data were available for 314 patients (50.3% male). Mean age was 64.0 years ($SD = 11.9$) and mean education was 12.5 years ($SD = 2.9$).

### 2.1.3. *Digit span sample*

To examine whether Digits Backward better differentiated frontal and nonfrontal stroke groups than Digits Forward, we excluded data for 141 patients because they had both frontal and nonfrontal lesions. In addition, we excluded data for 18 frontal and 54 nonfrontal patients because they had four or more lesions. After these exclusions, data were available for 52 frontal and 175 nonfrontal patients. Frontal and nonfrontal groups were similar in years of age (frontal: $M = 65.2$, $SD = 12.5$; nonfrontal: $M = 66.8$, $SD = 11.9$: $t(224) = 0.81$, $p > 0.05$), years of education (frontal: $M = 12.7$, $SD = 3.2$; nonfrontal: $M = 12.3$, $SD = 2.5$; $t\ (66.51) = 0.91$, $p > 0.05$), gender (frontal: 51.9% male: nonfrontal 48.6% male: $\chi^2(1) = 0.18$, $p > 0.05$), and ethnicity (frontal: 57.7% Caucasian: nonfrontal: 54.9% Caucasian: $\chi^2(4) = 4.67$, $p > 0.05$).

To examine the relationship between stroke severity (i.e., NIHSS score) and Digit Span performance regardless of lesion location, data were available for 440 patients (48.3% male). Mean age was 65.7 years ($SD = 11.94$) and mean education was 12.3 years ($SD = 2.8$).

### 2.1.4. *Generalizability*

The characteristics of patients were examined further to ensure the generalizability of our results. The proportion of frontal patients with available cognitive data (tested = 74; not tested = 36) was equivalent to that of nonfrontal patients (tested = 232; not tested = 96) ($\chi^2(1) = 0.47$, $p > 0.05$). To determine whether patients with cognitive data were similar to those for whom cognitive data were not available (but met exclusionary criteria), we conducted analyses of variance (ANOVA) with cognitive data availability (tested, not tested) and lesion location (frontal, nonfrontal) as between subjects factors on the following dependent measures: age, education, NIHSS scores, and number of lesions. There were no differences in education or number of lesions between patients with and without cognitive data, and availability of cognitive data did not interact with lesion location ($F < 1$, $p > 0.05$ in all instances). However, patients with cognitive data were slightly younger ($M = 64.5$, $SD = 11.9$) than patients without cognitive data ($M = 69.3$, $SD = 11.9$) ($F(1{,}422) = 8.82$, $p < 0.01$), although frontal and nonfrontal patients did not differ in this respect ($F(1{,}422) = 0.95$, $p > 0.05$). Patients with cognitive data had lower NIHSS scores ($M = 4.3$, $SD = 3.8$) than those without cognitive data ($M = 11.1$, $SD = 7.7$) ($F(1, 422) = 49.98$, $p < 0.001$), but frontal and nonfrontal patients did not differ in this respect ($F(1{,}422) = 0.08$, $p > 0.05$).

### 2.2. *Procedures*

#### 2.2.1. *Trail making test*

The Trail Making test was administered using standard testing procedures by an occupational therapist. Trails A was presented first, followed by Trails B. Patients were given a maximum time of 300 seconds to complete each condition of the test (i.e., Trails A and Trails B). The time in seconds to complete each condition and the logarithm of the time to complete each condition were used in the analyses.

#### 2.2.2. *Digit span*

The Digit Span test was administered using standard testing procedures by a speech pathologist. In the Digits Forward condition, patients listened as the pathologist read aloud a series of digits at a rate of 1 digit per second. Following presentation of the series, patients were asked to report the digits in the order presented. The digits ranged from 1 to 9, and the length of the series presented ranged from 2 to 9 digits with two trials at each series length (e.g., 3-7, 9-5, 3-7-2, 8-4-1, etc.). When patients missed both trials at a given series length, testing was discontinued. Patients then completed the Digits Backward condition. The procedure for this condition was identical to that for Digits Forward, except that following presentation of each series, patients were asked to report the digits in reverse order (e.g., presentation 6-2-9; report 9-2-6). Memory span scores for Digits Forward and Backward were recorded as the number of items in the longest series correctly recalled.

#### 2.2.3. *NIHSS*

The NIHSS [22] is widely used to assess stroke severity in the United States and abroad [24,25]. This is a 15-item scale that measures stroke-related neurological deficit. Items assess gaze, motor strength, sensory loss, language, ataxia, extinction/inattention, and level of consciousness. These items have a reliable factor structure and correlate with assessments up to three months later [26]. Previous research has shown that the NIHSS is associated with clinical dementia ratings, Barthel Index scores, Instrumental Activities of Daily Living, and Frenchay Activity Index performance [27, 28]. Further, the NIHSS is a predictor of decisions regarding discharge planning and rehabilitation [29,30].

## 3. Results

### 3.1. *Trail making test*

Following Stuss et al. [12], data from the Trail Making Test were analyzed using both the completion time in seconds from each condition and the logarithms of these times. Taking the logarithms produced a better match in the shapes of the distributions for the two conditions, as exemplified by a correlation of 0.62 between the completion times for Trails A and B and a correlation of 0.72 between the logarithms of the completion times for Trails A and B.

As a first analytic step, impairment on the Trail Making Test was examined by comparing the performance of frontal and nonfrontal groups to normative data based on years of age and education [31]. A z score greater than 1.5 reflected clinically significant impairment. Repeated measures ANOVA, with z score as the dependent variable and condition (Trails A, Trails B) and group (frontal, nonfrontal) as the independent variables revealed no significant main effect of group ($F(1{,}162) < 1.0$, $p > 0.05$) or condition ($F(1{,}162) < 1.0$, $p > 0.05$), and no significant interaction between group and condition, ($F(1{,}162) < 1.0$, $p > 0.05$). Further analyses revealed that on Trails A the z scores of frontal ($M = 3.6$, $SD = 5.6$, range $= -0.6$ to 29.3) and nonfrontal ($M = 4.1$, $SD = 6.4$, range $= -1.7$ to 29.3) groups did not differ ($t(162) = 0.16$, $p > 0.05$). Similarly, on Trails B, the z scores of frontal ($M = 3.9$, $SD = 4.0$, range $= -1.2$ to 16.4) and nonfrontal ($M = 4.8$, $SD = 5.3$, range $= -1.8$ to 25.0) groups did not differ ($t(162) = 1.04$, $p > 0.05$). Additional analyses also showed that there was no difference between groups in the number of patients with clinically significant im-

Table 1
Means (*SD*s) on Trail Making Test and Digit Span for frontal and nonfrontal groups

| Group | n | NIHSS *M* (*SD*) | Trails A s *M* (*SD*) | Trails B s *M* (*SD*) |
|---|---|---|---|---|
| Frontal | 45 | 4.5 (2.7) | 87.9 (77.7) | 184.2 (92.3) |
| Nonfrontal | 122 | 3.4 (4.6) | 88.4 (76.1) | 189.3 (92.8) |
| Acute | | | | |
| Frontal | 20 | 4.6 (5.1) | 75.3 (66.8) | 157.8 (91.6) |
| Nonfrontal | 60 | 3.5 (2.8) | 94.1 (82.7) | 192.6 (98.4) |
| MCA Only | | | | |
| Frontal | 27 | 4.8 (4.7) | 89.1 (75.3) | 184.8 (97.1) |
| Nonfrontal | 122 | 3.4 (2.7) | 88.4 (76.1) | 189.3 (92.8) |
| **Group** | **n** | **NIHSS *M* (*SD*)** | **Digits Forward *M* (*SD*)** | **Digits Backward *M* (*SD*)** |
| Frontal | 52 | 4.8 (4.2) | 5.9 (1.1) | 3.4 (1.0) |
| Nonfrontal | 175 | 4.5 (4.2) | 5.8 (1.3) | 3.2 (1.4) |
| Acute | | | | |
| Frontal | 27 | 4.9 (4.6) | 5.9 (1.2) | 3.4 (1.1) |
| Nonfrontal | 90 | 4.6 (4.2) | 6.0 (1.2) | 3.2 (1.3) |
| MCA Damage | | | | |
| Frontal | 30 | 4.4 (3.6) | 5.3 (0.9) | 3.4 (0.9) |
| Nonfrontal | 175 | 4.5 (4.2) | 5.8 (1.3) | 3.2 (1.4) |

MCA = middle cerebral artery.

pairment on Trails A (frontal: 51%; nonfrontal: 54%: $\chi^2(2) = 0.16$, $p > 0.05$) and Trails B (frontal: 51%; nonfrontal: 54%; $\chi^2(2) = 0.16, p > 0.05$).

Of particular interest was whether Trails B (either completion time or log completion time) better differentiated between frontal and nonfrontal groups than Trails A (*M* and *SD* shown in Table 1). Although in absolute terms the NIHSS indicated more severe stroke for the frontal than nonfrontal group, this difference was not statistically significant ($t(55.58) = 1.57$, $p > 0.05$). Multivariate analysis of variance (MANOVA), using completion time and log completion time on Trails A and B as dependent variables and group (frontal, nonfrontal) as the independent variable, revealed no between-group difference in either completion time ($F(2{,}164) < 1.0, p > 0.10$) or log completion time ($F(2{,}164) < 1.0, p > 0.10$).

In the analysis just described, patients with acute lesions and patients with both acute and prior lesions were included. Next, we conducted analyses focusing on data from patients (20 frontal, 61 nonfrontal) with only acute lesions. Frontal and nonfrontal groups did not differ in NIHSS ($t(22.96) = 0.90$, $p > 0.05$). MANOVAs, using completion time and log completion time on Trails A and B as dependent variables and group as the independent variable, revealed no between-group differences in either completion time ($F(2, 78) = 0.97$, $p > 0.05$) or log completion time ($F(2, 78) = 0.89$, $p > 0.05$).

Table 2
Correlations between performance and NIHSS in frontal and nonfrontal patients

| | Frontal | Nonfrontal |
|---|---|---|
| Trails A (s) | 0.450*** | 0.302* |
| Trails B (s) | 0.377* | 0.281* |
| Trails A (log) | 0.310* | 0.310* |
| Trails B (log) | 0.269* | 0.269* |
| Digits Forward | −0.079 | −0.177* |
| Digits Backward | −0.099 | −0.220* |

*$p < 0.05$; ***$p < 0.001$.

Thus far analyses included patients with lesions affecting anterior cerebral artery and middle cerebral artery distributions to capture frontal lobe lesions. However, the dorsolateral prefrontal cortex is more often associated with impairment in executive abilities [12]. To target the dorsolateral region, we conducted additional analyses in which frontal patients with lesions in the anterior portion of the anterior cerebral artery were excluded. The resulting sample included 27 dorsolateral frontal and 122 nonfrontal patients. Dorsolateral frontal patients did not have higher NIHSS scores than nonfrontal patients ($t(29.93) = 1.52$, $p > 0.05$), and there was no between-group difference in either completion time or log completion time on Trails A or B ($F(2{,}146) < 1.0, p > 0.10$ in both instances).

The next issue evaluated was whether performance on Trails B was more strongly associated with stroke severity (regardless of lesion location) than Trails A (see correlations in Table 2). To address this issue, we examined the correlations between NIHSS scores and completion time and log completion time on Trails A and B. Regardless of the measure used (completion time or log completion time), both conditions of the Trail Making Test were positively correlated with stroke severity (completion time Trails A: $r = 0.41$; completion time Trails B: $r = 0.34$; log completion time Trails A: $r = 0.41$; log completion time Trails B: $r = 0.33$). The correlation between the NIHSS and Trails A was significantly greater than the correlation between the NIHSS and Trails B (for both measures, $p < 0.001$). Controlling for completion time on Trails A greatly reduced the correlation between completion time on Trails B and NIHSS score ($pr = 0.11$, $p < 0.05$), and controlling for log completion time on Trails A eliminated the correlation between log completion time on Trails B and NIHSS score ($pr = 0.05$, $p > 0.05$). Further, the correlations between Trails A and NIHSS scores did not differ between frontal and nonfrontal groups regardless of the measure used (completion time: $z = 0.96$; log completion time: $z = 1.03$; $p > 0.10$ in both instances). More importantly, the correlations between Trails B and NIHSS scores for frontal and nonfrontal groups did not differ significantly (completion time: $z = 0.60$; log completion time: $z = 0.58$; $p > 0.10$ in both instances).

### 3.2. *Digit span*

Impairment on Digit Span was first established by comparing the performance of frontal and nonfrontal groups to normative data based on years of age [32]. A z score less than $-1.5$ reflected clinically significant impairment. Repeated measures ANOVA, with z score as the dependent variable and condition (Digits Forward, Digits Backward) and group (frontal, nonfrontal) as independent variables revealed no significant main effect of group ($F(1{,}224) < 1.0$, $p > 0.05$). Further analyses revealed that Digits Forward z scores for the frontal ($M = -0.2$, $SD = 0.1$, range $= -2.3$ to 1.5) and nonfrontal ($M = -0.3$, $SD = 0.1$, range $= -3.2$ to 2.5) groups did not differ ($t(162) = 0.16, p > 0.05$). Similarly, on Digits Backward, the z scores of frontal ($M = -0.8$, $SD = 0.1$, range $= -2.1$ to 1.4) and nonfrontal ($M = -1.0$, $SD = 0.1$, range $= -2.1$ to 1.4) groups did not differ ($t(162) = 1.04, p > 0.05$). There was a significant main effect of condition, with lower z scores on Digits Backward than Forward ($F(1{,}224) = 73.11$, $p < 0.001$). However, there was no interaction between condition and group ($F(1{,}224) < 1.0$, $p > 0.05$), indicating that the decrease in z scores on Digits Backward relative to Digits Forward was similar for frontal and nonfrontal groups. Additional analyses showed that there was no difference between groups in the number of patients with clinically significant impairment on Digits Forward (frontal: 8%; nonfrontal: 14%; $\chi 2_{\ (2)} = 1.68, p > 0.05$) or Backward (frontal: 15%; nonfrontal: 27%; $\chi^2(2) = 3.25, p > 0.05$).

Of particular interest was whether Digits Backward better differentiated between frontal and nonfrontal groups ($M$ and *SD* shown in Table 1). There was no significant difference between frontal and nonfrontal groups with respect to NIHSS ($t(225) = 0.36$, $p > 0.10$). A MANOVA, using longest series recalled on Digits Forward and Backward as dependent variables and group as the independent variable, revealed no between-group difference ($F(2{,}224) < 1.0, p > 0.10$).

As with the Trail Making Test, we conducted further analyses using data from patients (27 frontal, 90 nonfrontal) with only acute lesions. A MANOVA, with the longest series recalled on Digits Forward and Backward as the dependent variables and group as the independent variable, revealed no between-group difference ($F(2, 114) < 1.0, p > 0.10$).

As in our analyses of the Trail Making Test, we targeted the dorsolateral prefrontal cortex by excluding patients with lesions in the anterior portion of the anterior cerebral artery. The resulting sample included 30 frontal and 175 nonfrontal patients. There was no between-group difference in Digit Span ($F(2, 202) = 1.74$, $p > 0.10$), replicating our results from the larger sample.

The next issue evaluated was whether performance on Digits Backward was more strongly associated with stroke severity (regardless of lesion location) than Digits Forward. To address this issue, we examined the correlations between NIHSS scores and the longest series recalled on Digits Forward and Backward (see Table 2). Both conditions of Digit Span were negatively correlated with NIHSS (Digits Forward: $r = -0.14$, $p < 0.05$; Digits Backward: $r = -0.28$, $p < 0.001$). It should be noted that the correlation between NIHSS scores and Digits Backward was significantly greater than the correlation between NIHSS scores and Digits Forward ($t(437) = 3.05$, $p < 0.001$). Controlling for performance on Digits Forward did not eliminate the correlation between Digits Backward and NIHSS score ($pr = -0.24, p > 0.05$).

Although performance on Digits Forward was significantly correlated with NIHSS in the nonfrontal but not frontal group, the magnitudes of the correlations were similar ($z = 0.25, p > 0.05$). As such, the difference with respect to significance may simply reflect the larger size of the nonfrontal group. Moreover, performance on Digits Backward was significantly correlated with NIHSS in only the nonfrontal group (nonfrontal: $r = -0.22$, $p < 0.01$; frontal: $r = -0.09$, *ns*). However, the correlations between performance on Digits Backward and stroke severity NIHSS for the frontal and nonfrontal groups did not differ significantly ($z = 0.34$, $p > 0.05$). Again, this is probably due to differences in sample size (i.e., larger nonfrontal sample) and the minimal level of Digit Span impairment for both groups (leaving little room for variation).

## 4. Discussion

The major goal of the present study was to determine whether performance on the Trail Making Test and Digit Span differentiated between patients with frontal and nonfrontal lesions associated with stroke and whether stroke severity, as measured by the NIHSS, was associated with performance on these measures. With respect to the Trail Making Test, frontal and nonfrontal patients were equally impaired on both Trails A and B. Further, both frontal and nonfrontal groups were impaired on the Trail Making Test relative to normative data. Additionally, the level of impairment on Trails B was comparable to that on Trails A and did not differ by lesion location. In terms of stroke severity, performances on Trails A and B were equally correlated with NIHSS scores in both frontal and nonfrontal patients. Previous studies indicated that both Trails A and B are sensitive to brain damage [7–9], which is consistent with our finding that performance on Trails A was as strongly correlated with stroke severity as performance on Trails B. Moreover, controlling for completion time on Trails A greatly reduced the correlation between stroke severity and completion time on Trails B. Further, controlling for log completion time on Trails A eliminated the correlation between stroke severity and log completion time on Trails B. These results suggest that Trails B does not provide any more information regarding stroke severity than does simply assessing performance on Trails A.

With respect to Digit Span, although both frontal and nonfrontal groups performed more poorly on Digits Backward than Digits Forward, neither group showed impaired performance on Digit Span relative to normative data. In terms of stroke severity, Digits Forward was only weakly correlated with stroke severity, whereas a stronger correlation was observed for Digits Backward. In past research Digits Forward has been reported to be less sensitive to brain damage than Digits Backward [16,21,35], and our results are consistent with this finding. It should be noted, however, that this was the case only for nonfrontal patients.

Taken together, our findings lend no support to the widely held assumption that Trails B and Digits Backward are more sensitive to frontal than nonfrontal brain lesions than Trails A and Digits Forward. This was the case regardless of whether we examined the performance of patients with any lesions affecting the frontal lobes, or restricted our analyses to patients with lesions limited to dorsolateral prefrontal regions associated with executive abilities. In addition, the pattern of findings remained the same when we examined the performance on patients with only acute lesions. This pattern of results was also obtained by Davidson et al. [13] who did not find performance differences between patients with and without dorsolateral damage on the Trail Making Test, verbal fluency, or the Wisconsin Card Sorting Test.

Alexander and Stuss [33] commented on the apparent lack of correspondence between results of studies of executive abilities using neuroimaging in healthy adults (which show greater frontal activation during executive conditions of executive tasks) versus patients with brain damage. Specifically, they noted the limitations of traditional tests of executive abilities, arguing that these tests were not actually designed to elucidate structure/function relationships. Although inconsistent with conventional wisdom, our findings provide support for their position. Other studies have also found no difference between patients with frontal and nonfrontal brain damage across the different conditions of the Trail Making Test and Digit Span [14,15,34,35]. Thus, it appears that the different conditions of the Trail Making Test and Digit Span are inadequate to capture functional impairments specific to frontal lobe lesions.

Our findings of robust correlations between Trails A and Trails B and between Digits Forward and Backward also suggest that the putatively executive and nonexecutive conditions are largely redundant. This is particularly true for the Trail Making Test, in which controlling for Trails A greatly reduced the correlation between stroke severity and Trails B, whereas the correlation between stroke severity and Digits Backward remained significant after controlling for Digits Forward. Importantly, our findings indicated that the Trail Making Test as a whole was more sensitive to stroke severity than the Digit Span test. Overall, however, the Trail Making Test and Digit Span, although useful for assessing stroke severity, contribute little in terms of assessing the specific nature of impairment.

It is also important to note that, unlike most prior studies of executive abilities in patients with stroke, patients in our study were assessed within 72 hours of hospital admission. In contrast, the patients studied by Leskelä et al. [15], for example, were assessed approximately 3.4 months following the occurrence of stroke. Nevertheless, our findings are consistent with theirs and extend these results to the Digit Span test. Thus, the timing of our assessments seems unlikely to be the reason why our results are inconsistent with the conventional wisdom regarding executive abilities and lesion location.

The importance of the timing of our assessments, however, is that they were administered at a point when they could potentially guide decisions regarding discharge planning and subsequent treatment. For example, rehabilitation specialists typically make their decisions regarding the implementation of physical, occupational, or speech therapies within the first several days of a patient's hospitalization. Our findings suggest that neuropsychologists who assist in making these and other crucial decisions must be aware of the limitations of traditional measures of executive abilities.

## Acknowledgments

The authors would like to thank the occupational therapists, speech pathologists, and nurse coordinators of the Stroke Management and Rehabilitation Team at Barnes-Jewish Hospital who collected the data analyzed here and Jack Baty, the data manager, from the Division of Biostatistics at Washington University. This research was supported by the James S. McDonnell Foundation, the National Institute on Aging Grant T32-AG00030-34, and the NINDS Washington University SPOTRIAS.
